# Prevention of hyperoxia-induced bronchial hyperreactivity by sildenafil and vasoactive intestinal peptide: impact of preserved lung function and structure

**DOI:** 10.1186/1465-9921-15-81

**Published:** 2014-08-13

**Authors:** Dorottya Czövek, Ferenc Peták, Yves Donati, Xavier Belin, Jean-Claude Pache, Constance Barazzone Argiroffo, Walid Habre

**Affiliations:** Department of Medical Physics and Informatics, University of Szeged, Szeged, Hungary; Department of Anesthesiology, Anesthesiological Investigation Unit, Pharmacology and Intensive Care, University of Geneva, Geneva, Switzerland; Department of Pediatrics, Pediatric Pulmonology Unit, Geneva Children’s Hospital, Geneva, Switzerland; Department of Pathology and Immunology, University of Geneva, Geneva, Switzerland; Pediatric Anesthesia Unit, Geneva Children’s Hospital, 6, Rue Willy Donzé, CH-1205 Geneva, Switzerland

## Abstract

**Objective:**

Hyperoxia exposure leads to the development of lung injury and bronchial hyperreactivity (BHR) via involvement of nitric oxide (NO) pathway. We aimed at characterizing whether the stimulation of the NO pathway by sildenafil or vasoactive intestinal peptide (VIP) is able to prevent the hyperoxia-induced development of BHR. The respective roles of the preserved lung volume and alveolar architecture, the anti-inflammatory and anti-apoptotic potentials of these treatments in the diminished lung responsiveness were also characterized.

**Materials and methods:**

Immature (28-day-old) rats were exposed for 72 hours to room air (Group C), hyperoxia (>95%, Group HC), or hyperoxia with the concomitant administration of vasoactive intestinal peptide (VIP, Group HV) or sildenafil (Group HS). Following exposure, the end-expiratory lung volume (EELV) was assessed plethysmographically. Airway and respiratory tissue mechanics were measured under baseline conditions and following incremental doses of methacholine to assess BHR. Inflammation was assessed by analyzing the bronchoalveolar lavage fluid (BALF), while biochemical and histological analyses were used to characterize the apoptotic and structural changes in the lungs.

**Results:**

The BHR, the increased EELV, the aberrant alveolarization, and the infiltration of inflammatory cells into the BALF that developed in Group HC were all suppressed significantly by VIP or sildenafil treatment. The number of apoptotic cells increased significantly in Group HC, with no evidence of statistically significant effects on this adverse change in Groups HS and HV.

**Conclusions:**

These findings suggest that stimulating the NO pathway by sildenafil and VIP exert their beneficial effect against hyperoxia-induced BHR via preserving normal EELV, inhibiting airway inflammation and preserving the physiological lung structure, whereas the antiapoptotic potential of these treatments were not apparent in this process.

## Introduction

Prolonged exposure to excessive concentration of oxygen is often applied in clinical practice to enhance oxygenation in the presence of compromised gas exchange. While the adverse pulmonary consequences of oxygen-toxicity have been well established, hyperoxia exposure is required in clinical situations involving severely compromised oxygenation, such as in newborn infants with premature birth [1–4] or in patients with acute lung injury [5]. Since a prolonged excessive oxygen concentration poses the risk of long-term adverse alterations in the lung architecture and pulmonary function [6], prevention of hyperoxia-induced lung injury is still an issue with major importance.

Hyperoxia provokes lung injury by inducing a primary massive necrosis of capillary endothelial cells and hence the loss of capillaries [7–9], which is followed by apoptosis/necrosis of the epithelial cells. All these adverse changes alter the epithelial tight junctions with a subsequent increase in alveolar-capillary barrier permeability leading to pulmonary edema and induction of the inflammatory cascade [10]. The chronic presence of all these pathophysiological changes may lead to airway and vascular remodeling in the lungs, with subsequent pulmonary hypertension [2, 11–14], fibrosis and the development of irreversible changes in the lung structure [15–18]. All these pathways contribute to the hyperoxia-induced development of bronchial hyperreactivity (BHR) [13, 14, 16, 17, 19], which plays a major role in the frequent development of respiratory adverse events due to the enhanced lung responsiveness to exogenous constrictor stimuli.

Previous studies demonstrated the deleterious effects of hyperoxia on nitric oxide (NO) production in the lungs via hyperoxia-induced epithelial and endothelial cell injuries [20–22]. Therefore, the resulting imbalance between the relaxation and constriction regulation of the airway smooth muscle may be responsible for the adverse lung functional and structural changes following hyperoxia. Thus, enhancing NO production in the lungs may counteract the elevated smooth muscle tone via a direct effect [23] or prevent against the development of chronic lung inflammation and subsequent airway remodeling [24]. Similar to NO, vasoactive intestinal peptide (VIP), which is also an inhibitory relaxant mediator of the non-adrenergic, non-cholinergic nervous pathway, may also exhibit dual bronchodilatory and anti-inflammatory potential [25–27]. Thus, we aimed at investigating whether the restoration of the NO-dependent processes with a phosphodiesterase type 5 (PDE5) inhibitor, such as sildenafil, or with VIP provides an effective protection against the development of BHR in the acute phase of hyperoxia. Furthermore, we also intended to clarify whether the preserved lung structure, or the anti-inflammatory and the anti-apoptotic potentials of these treatments are responsible for the diminished lung responsiveness.

## Materials and methods

### Treatments and hyperoxia exposures

The experimental protocol was approved by the Experimental Ethics Committee of the University of Geneva and the Animal Welfare Committee of the Canton of Geneva (1051/3691/II). Weanling male Sprague–Dawley rats (80–125 g) were exposed to either hyperoxia (>95% O_2_) or normoxia (room air, Group C, n = 8) for 72 hours. The hyperoxia-exposed animals were randomized to 3 groups: no additional treatment (Group HC, n = 8), the daily oral administration of sildenafil (20 mg/day; Pfizer, Zürich, Switzerland Group HS, n = 7) or the daily i.p. injection of VIP (150 μg/day; Sigma-Aldrich, Buchs, Switzerland Group HV, n = 8), in both cases starting simultaneously with the commencement of the exposure to hyperoxia and lasting until the day prior to the experiments. During the 72-hour oxygen or room air exposure the rats were kept in a 98-liter sealed normobaric Plexiglas chamber (Elega, Geneva, Switzerland). The chamber was opened for a short time (<5 min) to allow delivery of the daily treatment. The oxygen and CO_2_ levels were checked twice a day (Datex, Helsinki, Finland). The CO_2_ level in the box was maintained below 1% by using a CO_2_ absorber (Sodasorb, Asid Bonz GmbH, Herrenberg, Germany). Food and water were available *ad libitum*.

### Experimental protocol

The rats were first anesthetized with an i.p. injection of chloral hydrate (5%, 350 mg/kg; Sigma Aldrich, Switzerland), then tracheostomized and the distal trachea was cannulated with a polyethylene cannula (16 G, 13 mm long; AMSINO Medical, Shangai, China). The animals were mechanically ventilated (model 683; Harvard Apparatus, South Natick, MA, USA) with a tidal volume of 8 ml/kg, a respiratory rate of 110 breaths/min and a positive end-expiratory pressure (PEEP) of 2.5 cmH_2_O. Anesthesia was maintained with the repeated administration of 50 mg of chloral hydrate every hour. After completion of the end-expiratory lung volume (EELV) measurements, a femoral artery was cannulated and attached to a pressure transducer (model 156 PCE 06-GW2; Honeywell, Zürich, Switzerland) to allow monitoring of the systemic blood pressure. At the beginning and at the end of the experiment, the arterial line was also used for blood gas analysis (model 505; Acid Base Laboratory, Copenhagen, Denmark). The femoral vein was also catheterized for drug delivery. Since application of the forced oscillation technique (FOT) to assess the respiratory mechanics requires apnea, atracurium (GlaxoSmithKline, Münchenbuchsee, Switzerland) was administered i.v. before the measurements in order to prevent the spontaneous breathing. This line was used for the methacholine (MCh) provocation (Bichsel AG, Interlaken, Switzerland). At the end of the surgical preparation, a deep inspiration to a pressure of 30 cmH_2_O was applied before the first FOT measurement so as to standardize the volume history. The mechanical ventilation was then paused at end-expiration, and 4–6 6-s-long recordings were collected at 1-min intervals between each measurement under baseline conditions. To assess the lung responsiveness MCh was infused i.v. in incremental doses from 4 to 8 and 16 μg/kg/min. The development of stable bronchoconstriction (airway resistance values were within 5%) required 5 min. Three forced oscillatory data recordings were collected and ensemble-averaged under a steady-state conditions 6 min after the onset of MCh provocation at each infusion level. After the last dose, a 15-min period was allowed for the rat to recover from the bronchoconstriction and 3 further Zrs recordings were made and ensemble-averaged.

At the end of the experiments, the right lung was clamped near the bifurcation and bronchoalveolar lavage fluid (BALF) was collected from the left lung 15 min after the last Zrs measurements in order to assess lung inflammation. For histological analyses, the right lung was then instilled via the trachea with 4% formaldehyde at a hydrostatic pressure of 20 cmH_2_O while the left lung was clamped. A piece measuring ~0.5 cm^3^ was excised and embedded in Optimal Cutting Temperature compound, frozen in liquid nitrogen vapor-cooled 2-methylbutane (Fluka Chemie GmbH, Buchs, Switzerland) and kept at -80°C until cryosection and TUNEL analysis for apoptosis quantification.

Intratracheal pressure, systemic blood pressure, ECG and rectal temperature were monitored throughout the experiments (Biopac, Santa Barbara, CA, USA). Body temperature was maintained at 37 ± 0.5°C through use ofa homeothermic blanket system (Homeothermic monitor; Harvard Apparatus, Edenbridge, UK).

The body weight and the basic blood gas parameters of the rats included in the experimental groups at the beginning of the experiments are demonstrated on Table 1. The retarded BW in the Group HS is most likely a consequence of the regular gavage. The higher HCO_3_ in the animals of Group HC can be attributed to the compensation of gas exchange abnormalities leading to CO_2_ accumulation and chronic respiratory acidosis during the hyperoxia exposure. Both treatments applied in the animals in Groups HS and HV were able to maintain normal gas exchange.

**Table 1:** **Anthropometric and blood gas data**

	Group C	Group HC	Group HS	Group HV
**BW (g)**	102.9 ± 14.5	96.5 ± 6.6	87 ± 5.9 ^*^	98.3 ± 8.9
**pH**	7.43 ± 0.07	7.39 ± 0.09	7.44 ± 0.1	7.41 ± 0.11
**PCO**_**2**_ **(Kpa)**	5.09 ± 0.8	5.97 ± 1.2 ^*^	5.12 ± 1.7	5.06 ± 1.58
**HCO**_**3**_ **(meq/l)**	24.2 ± 1.8	30.5 ± 4.2 ^*^	28.3 ± 6.5	23.1 ± 2.29

Mean ± SD of the body weight (BW), pH, partial pressure of CO_2_ and the bicarbonate level (HCO_3_) in the arterial blood sample obtained at the beginning of the experiment (day 4). *: p < 0.05 vs. Group C.

### EELV measurements

EELV was measured at a PEEP of 2.5 cmH_2_O by using a whole-body plethysmograph (620 ml), as detailed previously [28]. Briefly, the rat was placed in a supine position in a sealed Plexiglas chamber. The tracheal cannula was connected to the respirator and also to a pressurized (2.5 cmH_2_O) loudspeaker chamber. Before the measurement, the mechanical ventilation was paused, the plethysmograph was opened to the atmosphere and the trachea was opened to the loudspeaker chamber to equilibrate the lungs to a pressure of 2.5 cmH_2_O. The airway opening and the plethysmograph box were then closed until the first few breathing efforts generated by the animal against the closed trachea. Six-to-eight breathing maneuvers were recorded for 10 s by measuring the tracheal (Ptr) and box (Pbox) pressure changes. The recordings of Pbox were then corrected for the thermal properties of the plethysmograph. Via Boyle’s law, EELV was calculated from the relationship between the corresponding changes in Ptr and Pbox [28].

Reliable EELV measurements were obtained in a subgroup of animals, in view of the fact that the small box pressure signals were recorded initially with the use of a plethysmograph originally designed for adult rats. The box volume was optimized for the size of the weanling rats only after this technical problem had been recognized.

### Airway and tissue mechanics and bronchial challenge

The input impedance of the respiratory system (Zrs) was estimated by FOT, as described in detail previously [29]. Briefly, the tracheal cannula was connected to a loudspeaker-in-box system generating a composite signal containing 23 components at low frequencies (0.5-20.75 Hz). The forcing signal was driven through a 100-cm-long and 2-mm-ID polyethylene wave-tube into the trachea during 6-s apneic periods. Two identical pressure transducers (Model 33NA002D; ICSensors, Malpitas, CA, USA) were used to measure the lateral pressures at the loudspeaker and at the tracheal end of the wave-tube. Zrs was calculated by applying the transmission line theory [30]. A model including frequency-independent resistance (Raw), inertance (Iaw) and a constant-phase tissue compartment with tissue parameters for damping (G) and elastance (H) was fitted to the ensemble-averaged Zrs spectra [31].

Data were corrected for body weight (BW). Zrs data were measured under baseline conditions and following i.v. methacholine (MCh) provocation (4, 8, 16 μg/kg/min) to assess the lung responsiveness. The equivalent dose causing a 50% increase in Raw (ED_50_) was calculated by linear interpolation.

### Bronchoalveolar lavage

After completion of the last FOT measurements, BALF was collected in order to analyze the inflammatory response to hyperoxia in the lungs. The lungs were instilled with 3 ml of PBS (37°C) via the tracheal cannula at a pressure of 20 cmH_2_O. From the withdrawn BALF, the total cell number was counted in a Neubauer hemocytometer by using the trypan blue exclusion method. BALF was then centrifuged at 200 × g for 10 min at 4°C. The BALF supernatant was collected to assess the protein content (PIERCE BCA assay kit; Rockford, IL, USA). The cell pellet was resuspended at 10^6^ cells/ml in PBS containing 1% BSA, and alveolar cell repartition was determined on cytospin after May-Grünwald Giemsa staining.

### Biochemical and histological analyses

The left lung was clamped, removed and preserved in Optimal Cutting Temperature compound at -80°C. Terminal deoxynucleotidyl transferase (TdT)-mediated deoxyuridinetriphosphate (dUTP) nick end-labeling (TUNEL) was performed on lung cryosections according to the protocol for the In Situ Cell Death Detection Fluorescein Kit (Roche, Mannheim, Germany). Briefly, 6-μm lung slices were fixed with 4% formaldehyde for 10 min at room temperature and next permeabilized for 2 min with 0.1% Triton X-100 in 0.1% sodium citrate at 4°C. The slices were then incubated with the labeling mix containing TdT and fluorescein-labeled dUTP for 1 hour at 37°C.

Fluorescent staining of total nuclei was performed with 250 ng/ml 4′,6-diamidino-2-phenylindole (Sigma) for 5 min at room temperature. The slides were mounted with FluorSave Reagent (Calbiochem, Nottingham, UK). Images for analysis were acquired by confocal microscopy (LSM 510 Meta; Carl Zeiss MicroImaging GmbH, Jena, Germany) and cells were counted by using MetaFluor Fluorescence Ratio Imaging Software (version 7.7.6; Molecular Devices, Sunnyvale, USA). The percentage ratio of the number of apoptotic nuclei from the total number of nuclei was calculated.

The right lung was fixed with 4% formaldehyde under a pressure of 20 cmH_2_O and embedded in paraffin. The 6-μm sections were stained with hematoxylin/eosin. Full-section images were captured with a MIRAX MIDI system (Carl Zeiss MicroImaging GmbH). Digitalized images were analyzed by using Panoramic Viewer software (3DHISTECH, Budapest, Hungary). Edema manifestation was quantified by relating the sum of edema surface area around the bronchi and vasculature to the total surface area of the lung tissue. Lung alveolar geometric structure and density by mean linear intercept (MLI) measurement was assessed by using the Image J software (version 1.46r, NIH, Bethesda, USA).

### Statistical analysis

Data are presented as means ± SEM. The Shapiro-Wilk test was used to test data for normality. The mechanical parameters were normally distributed. Accordingly, two-way repeated measures analysis of variances (ANOVA), with variables of MCh dose (baseline, 4, 8, 16 μg/kg/min) and groups (C, HC, HS and HV), was used to evaluate the effects of hyperoxia on the lung responsiveness to the bronchoconstrictor provocation.

To analyze the effects of hyperoxia on the inflammatory response (total cell number, percentage of PMN cells and protein), or on the functional and structural changes (ED_50_, EELV, MLI and edema) and on the apoptosis, one-way ANOVA with the post hoc Holm-Šidák test was used in the event of normal distribution. When the distribution was not normal, ANOVA on ranks was applied for the statistical analysis. p < 0.05 was considered statistically significant.

## Results

### Hyperoxia-induced lung hyperresponsiveness and elevated lung volume

**Figure 1:**
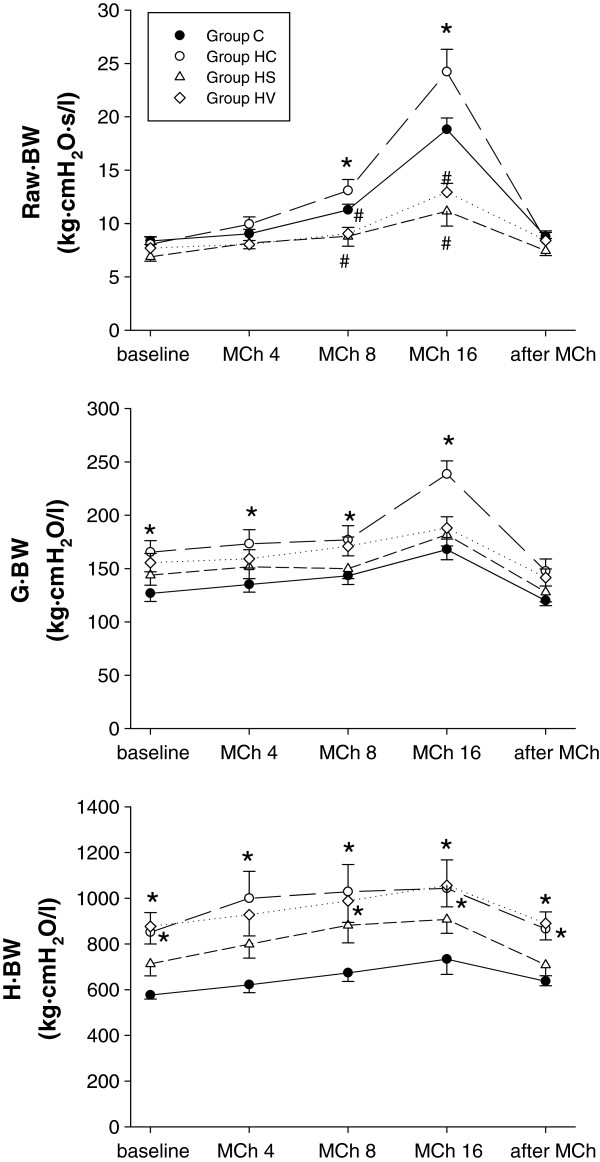
**Airway and respiratory tissue mechanical parameters normalized to body weight (airway resistance: Raw · BW, tissue damping: G BW, tissue elastance: H BW) under baseline conditions, during methacholine challenges (MCh 4-8-16) and after the bronchoprovocation tests (after MCh) in rats exposed to room air (Group C) subjected to hyperoxia without any additional treatment (Group HC) and hyperoxic rats treated either with sildenafil (Group HS) or VIP (Group HV).** *: p < 0.05 vs. Group C, ^#^: p < 0.05 vs. Group HC.

Figure 1 presents the results of the respiratory mechanical measurements made in the protocol groups under baseline condition and following provocations with incremental i.v. doses of MCh. There was no evidence of a statistically significant difference in the baseline values of Raw · BW following hyperoxia exposure, whereas G · BW was significantly elevated in Group HC (p < 0.01 vs. Group C), and H · BW was also higher in Groups HC (p < 0.05) and HV (P < 0.01). The development of airway hyperresponsiveness was apparent from the significantly greater responses to MCh in the rats in Group HC (p < 0.05 and p < 0.001, at the second and third doses of MCh vs. Group C, respectively), whereas a significant reduction in the lung responsiveness to MCh was obvious in the hyperoxic rats treated with sildenafil (Group HS, P < 0.001 at the third dose of MCh vs. Group C) or VIP (Group HV, p < 0.001 at third dose of MCh vs. Group C). The enhanced lung responsiveness in Group HC was also manifested in the significantly greater increases in G · BW in response to MCh (p < 0.005), whereas there was no evidence of an enhanced response in this parameter following either sildenafil or VIP treatment. No statistically significant treatment-dependent differences were observed in the slight MCh-induced elevations in H · BW. All mechanical parameters exhibited a complete recovery by 15 min after the last MCh dose, with no statistically significant difference from their initial values.

**Figure 2:**
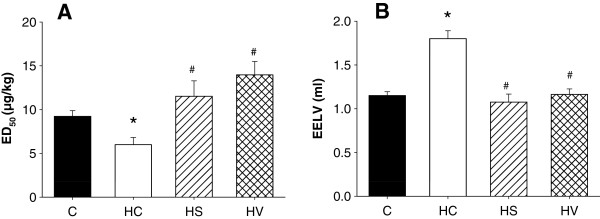
**Equivalent doses of methacholine required to cause a 50% elevation in airway resistance (ED**_**50**_ **, panel A) and the end-expiratory lung volumes (EELV, panel B) measured in rats exposed to room air (Group C) subjected to hyperoxia without any additional treatment (Group HC) and hyperoxic rats treated either with sildenafil (Group HS) or VIP (Group HV).** *: p < 0.05 vs. Group C, ^#^: p < 0.05 vs. Group HC.

The difference due to hyperoxia in the airway responsiveness to MCh is also reflected in the statistically significantly decreased ED_50_ value (Figure 2A) in Group HC as compared with Group C (p < 0.01). Both sildenafil and VIP treatments significantly elevated this hyperoxia-induced lowering in ED_50_ (p < 0.01 and p < 0.001, respectively). EELV measurements were successfully made in subgroups of 5 rats in each of Groups C and HC, and of 4 animals in each of Groups HS and HV. Exposure to hyperoxia significantly increased EELV in the rats in Group HC (Figure 2B; p <0.001 vs. Group C, HS and HV), but this was blocked by the sildenafil and VIP treatments.

### Effects of treatments on hyperoxia-induced lung inflammation

**Figure 3:**
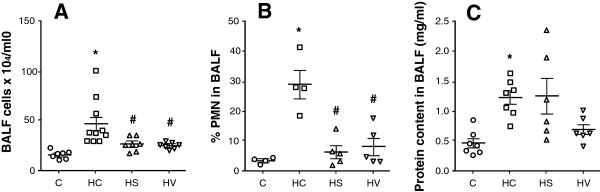
**Total cells (panel A), the repartition to polymorphonuclear cells (panel B) and the protein content (panel C) of the BALF obtained from rats exposed to room air (Group C) subjected to hyperoxia without any additional treatment (Group HC) and hyperoxic rats treated either with sildenafil (Group HS) or VIP (Group HV).** *: p < 0.05 vs. Group C, ^#^: p < 0.05 vs. Group HC.

The alterations in the cellular profile and the protein content of the BALF are outlined in Figure 3. Hyperoxia led to an increased number of inflammatory cells in the BALF collected from the animals in Group HC (Figure 3A, p < 0.001 vs. Group C and p < 0.01 vs. Groups HS and HV). The repartition of the cell types differed in Group HC from the other groups of rats; the percentage of polymorphonuclear cells was significantly increased (Figure 3B, p < 0.001 vs. Groups C, HS and HV). The protein content of the supernatant was significantly elevated in Group HC relative to Group C (Figure 3C, p < 0.001), while there was no evidence of a significant change in the sildenafil or VIP-treated animals as compared with Groups HC (Figure 3C).

### Effects of treatments on the hyperoxia-induced lung structural changes

**Figure 4:**
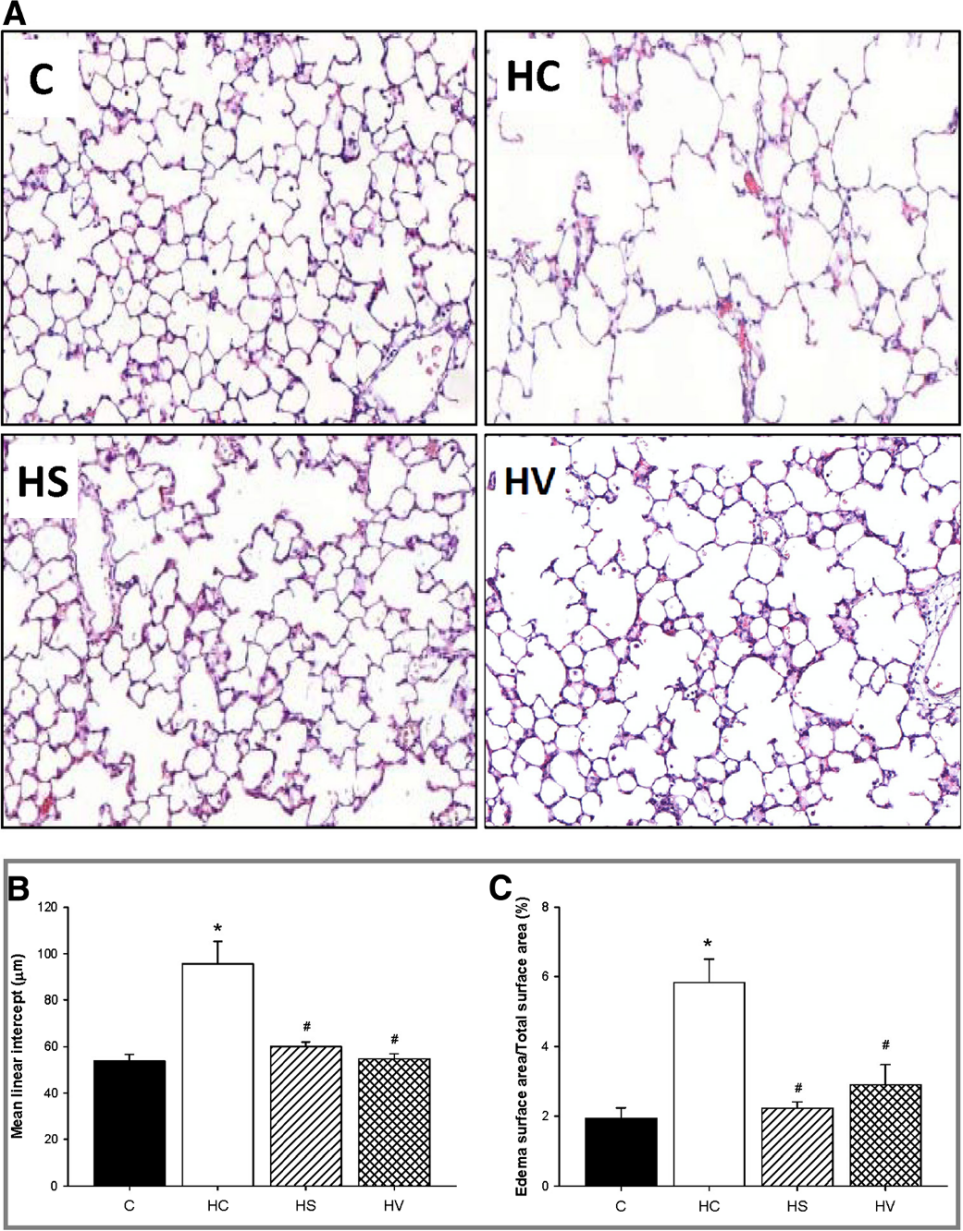
**Representative images of hematoxylin/eosin-stained lung sections (panel A) collected from control rats exposed to room air(C), rats subjected to hyperoxia without any additional treatment (HC), and hyperoxic rats treated with either sildenafil (HS) or VIP (HV).** Mean linear intercept values **(panel B)** and percentage of edema surface **(panel C)** were determined from the lung sections of the rats in the protocol groups. *: p < 0.05 vs. Group C, ^#^: p < 0.05 vs. Group HC.

The lung histological findings are depicted in Figure 4. The lung sections of untreated animals subjected to hyperoxia (Group HC, Figure 4A) revealed an aberrant alveolarization with decreased alveolar septation and consequently enlarged alveolar spaces in comparison with normoxia-exposed lungs (Group C, Figure 4A). These changes are expressed by the significantly increased MLI value (Figure 4B, p < 0.001 vs. Group C). When sildenafil (Group HS, Figure 4A) or VIP (Group HV, Figure 4A) treatment was applied concomitantly with hyperoxia, normal alveolarization was preserved, as indicated by the MLI values, which exhibited no statistically significant difference from those for the normoxia group (Figure 4B). The 72 hours of hyperoxia led to a vascular leakage, as revealed by the edema around the pulmonary vessels (Figure 4C). The ratio of the edema surface area to the total surface area of the lung sections was significantly greater in the non-treated animals exposed to hyperoxia than in the controls (p < 0.001) or in the hyperoxia-treated rats (p < 0.001 and p < 0.01 vs. Group HS and HV, respectively).

### Effects of treatments on the hyperoxia-induced apoptosis

**Figure 5:**
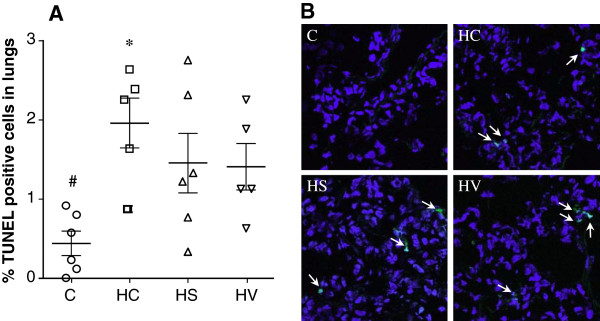
**Percentages of TUNEL-positive nuclei counted in lung cryosections (panel A) collected from rats exposed to room air (Group C) subjected to hyperoxia without any additional treatment (Group HC) and hyperoxic rats treated either with sildenafil (Group HS) or VIP (Group HV).** *: p < 0.05 vs. Group C, ^#^: p < 0.05 vs. Group HC. Representative images for TUNEL in cryosections from the studied groups are shown in panel **B**. Arrows indicate TUNEL-positive nuclei (green).

For each lung cryosection, the total number of nuclei and the number that were TUNEL-positive were counted in 10 independent randomized microscopic fields, representing at least 500 nuclei (506 to 1190); these results are presented in Figure 5. Exposure to hyperoxia induced a statistically significant increase in the percentage of positive nuclei in Group HC relative to that in Group C (p < 0.01). No evidence of statistically significant differences was observed in the sildenafil or VIP-treated rats as concern the apoptosis in the lung cells when it was compared to the untreated hyperoxic or normoxic animals.

## Discussion

The present study explored the lung function deterioration and the development of an enhanced airway responsiveness occurring in the acute phase of hyperoxia exposure. Our findings demonstrate no detectable effects of hyperoxia on the baseline airway function, whereas oxygen exposure induced deleterious changes in the baseline values of the parameters reflecting dissipative and elastic properties of the respiratory tissues, and marked elevations in the end-expiratory static lung volume. The results also demonstrated that the PDE5-inhibitor sildenafil and the NO-liberator VIP abolished the hyperoxia-induced development of airway hyperresponsiveness. These beneficial properties were associated with the abilities of both treatments to maintain normal EELV, preserving the normal alveolarization, exerting a beneficial effect on the inflammatory response and preventing the edema formation in the developing lung during hyperoxia exposure. However, none of the treatments proved beneficial in the inhibition of the hyperoxia-induced apoptosis.

The immature rat model was chosen in the present study, since hyperoxia exposure is often required in clinical situations involving severely compromised oxygenation, such as in infants [2, 32]. To address the prevention of BHR appropriately, respiratory mechanical measurements combined with provocation tests are inevitable. We intended to apply intravenous delivery of the provocation agonist, as this route of administration gives more representative picture about the overall lung response [33]. The need for securing venous and arterial lines for reliable respiratory mechanical measurements necessitated the use of weanling rats rather than much younger rat pups. Nevertheless, the findings obtained in our model can be also generalized to adult patients with acute lung injury, where a prolonged excessive oxygen exposure is also part of the therapy [5].

### Respiratory function and lung responsiveness

In accordance with earlier results when immature rats were exposed to oxygen for a short time [16], the basal Raw was not affected by hyperoxia exposure (Figure 1) and is also in line with the notion that Raw is determined primarily by the geometry of the central conducting airways, which remained unaffected [19, 34, 35]. An airway obstruction was detected in earlier studies when exposure to oxygen was applied for a prolonged period (e.g. 4 weeks), which was sufficient for airway remodeling to develop [36].

The 72-hour exposure to hyperoxia in the present study resulted in increases in the basal values of both viscoelastic tissue parameters (Figure 1; G · BW and H · BW), which accords with the results of previous studies, where a decreased compliance was found after oxygen toxicity [19, 36–38]. Since the hyperoxia-induced increases in G · BW and H · BW were proportional, the enhancement of ventilation heterogeneities was not likely to have played a role in these findings [39]. It seems more probable that perivascular and interstitial edema formation are responsible for these proportional elevations, this being substantiated by the histological findings (Figure 4C) and the elevated protein level in the BALF (Figure 3C). Nevertheless, other processes may also have been involved in the increase in H · BW, since an elevation in elastance was also observed in the VIP-treated rats, despite the lack of manifest edema. Such processes may be related to hyperventilation throughout the exposure, striving to compensate the hyperoxia and causing hypertrophy of the intercostal muscles, leading to stiffening of the entire respiratory system.

Our findings demonstrate the development of lung hyperresponsiveness at 72 hours after the onset of hyperoxia, manifested in a decreased ED_50_ value (Figure 2A), in accordance with the results of the few previous studies where bronchoprovocation tests were performed [16, 38, 40]. It is noteworthy that this functional abnormality is already present despite the lack of remodeling of the bronchial wall at this acute stage of oxygen toxicity [34].

While the presence of BHR was consistently established in earlier studies after hyperoxia exposures, the efficient prevention of this major pulmonary symptom has not been investigated. The main finding of the present study is that both sildenafil and VIP compensated and even overcompensated the enhanced lung responsiveness. To address the role of the mechanisms responsible for this finding in detail, alveolar structural and lung configurational changes, inflammatory alteration and biochemical profile were compared between the protocol groups.

### Role of lung morphological changes in the abolished BHR

In our hyperoxia model, the 72-hour oxygen exposure was sufficiently long for an abnormal alveolar structure to be observed in the developing lungs (Figure 4A). Compensation of the previously demonstrated decreased NO activity [20, 22], either through stimulation of the cyclic guanosine monophosphate-dependent effects by using sildenafil to activate vascular endothelial growth factor [41], or through enhancement of the NO production by VIP treatment was able to preserve normal alveolarization despite maintenance of the hyperoxic environment (Figure 4). Counting MLI confirmed that enlarged alveolar spaces were observed only in the non-treated oxygen-exposed animals; in the other groups, the MLI data were normal. These findings point to the involvement of the preserved physiological alveolar structure in the protection of the adverse lung functional changes following prolonged oxygen exposure and suggest its contribution to the abolishment of the hyperoxia-induced BHR.

### Role of preserved normal lung volume in the abolished BHR

While lung functional changes have been well established following hyperoxia-induced lung injury, and this damage has been reported to increase terminal air space size [42], we are unaware of any previous papers addressing the changes in the static volumes in a developing lung. Thus, our finding is the first to reveal that the changes in alveolar architecture were also reflected in the hyperoxia-induced EELV elevation in Group HC (Figure 2A). In this emphysematous condition, air is trapped in the enlarged alveolar spaces at end-expiration and the gas content of the chest is increased. Moreover, the small airways are likely to display a tendency to collapse during expiration due to their structural impairment, and their function may be further compromised by the decreased elastic support of the damaged lung parenchyma, which would be less able to keep the small airways open. Since the sildenafil and VIP prevented the enlargement of terminal airspaces, the physiological EELV in these animals is in line with the histological findings. This further confirms the abilities of these treatments to preserve physiological lung configuration during hyperoxia exposures.

### Role of anti-inflammatory potentials in the abolished BHR

Since the inflammatory cells appear in the first few hours of hyperoxia exposure [43], early inhibition of the inflammatory response might prevent the lung injury and thus, this pathway is expected to contribute to the development of BHR. As confirmed by our findings (Figure 3A, B), VIP was shown to protect alveolar epithelial cells against hyperoxia via decreasing neutrophil influx into the lung [27, 44]. Thus, treatments with sildenafil or VIP protect against the deleterious consequences of hyperoxia-induced lung inflammation by inhibiting polymorphonuclear cell infiltration into the alveolar compartment and reducing the consecutive tissue destruction. This beneficial profile of NO may be related to the down-regulation of the intracellular adhesion molecule and monocyte chemotactic protein-1 [45]. These findings indicate that the potent anti-inflammatory activities of sildenafil and VIP play a key role in the prevention of functional abnormalities, including the development of BHR, following acute hyperoxia exposures.

### Role of alveolo-capillary disruption in the abolished BHR

Elevated amounts of reactive oxygen species (ROS) are known to cause alterations in DNA and proteins and are the major contributors to alveolar cell death in hyperoxia [9, 46]. The alveolar cell death is an early event in hyperoxia-induced lung injury [9, 47]. In agreement with the literature finding, the number of apoptotic/necrotic cells in rat lung was elevated following exposure to hyperoxia for 72 hours. However, there was no evidence for sildenafil or VIP to prevent apoptosis and necrosis (Figure 5), suggesting that the preserved lung function and responsiveness in these animals are not related to the abilities of the these treatments to counteract the initial ROS injuries. Accordingly, the anti-apoptotic potential of sildenafil and VIP does not contribute to the abolished hyperoxia-induced BHR despite the presence of a close link between the apoptosis and BHR [48, 49].

### Other potential contributing mechanisms in the abolished BHR

A further potential explanation for the abolished BHR following hyperoxia exposures may be related to the direct effects of the treatments on the lung responsiveness. Since excessive NO production exerts a potent smooth muscle relaxation [23], this may have counteracted the MCh-induced constrictor stimuli, and subsequently diminished the airway responsiveness. This possible effect is due to the direct functional antagonism of the constrictor stimuli and thus, its efficiency may be independent from the presence of lung injury. Since the half-life of sildenafil is 3–6 hours [50] and it is even much shorter for VIP [51], and both treatments were terminated on the days before the experiment, this direct mechanism was unlikely to play a detectable role.

## Conclusions

The results of the present study demonstrate that enhancing the activity of NO-mediated pathways by sildenafil and VIP prevent the development of hyperoxia-induced BHR by preserving normal alveolarization, preventing lung volume enlargement, and inhibiting lung inflammation and edema formation. The lack of prophylactic activities of sildenafil and VIP against hyperoxia-induced apoptosis despite the abolished BHR suggests that this pathway did not contribute to the enhanced lung responsiveness. Our findings therefore point to the potential benefit of sildenafil or VIP treatment in the critical acute phase of hyperoxic lung damage by demonstrating their abilities not only to prevent the irreversible functional and structural changes in the lungs, but also to preserve physiological lung responsiveness.
